# Vital Pulp Therapy with Calcium-Silicate Cements: Report of Two Cases 

**DOI:** 10.22037/iej.2017.23

**Published:** 2017

**Authors:** Hengameh Ashraf, Afsaneh Rahmati, Neda Amini

**Affiliations:** a * Endodontic Department, Dental School, Shahid Beheshti University of Medical Science, Tehran, Iran;*; b *Endodontic Department, Dental School, Hamedan University of Medical Science, Hamedan, Iran; *; c *Dentist, Professional Trainee at University of Texas Health Science Center at Houston*

**Keywords:** Apexogenesis, Calcium-Silicate Cements, Dental Pulp, Pulpotomy, Vital Pulp Therapy

## Abstract

This article describes successful use of calcium-enriched mixture (CEM) cement and Biodentine in apexogenesis treatment in two 8-year-old patients, one with immature permanent molar diagnosed primarily with irreversible pulpitis and the other with partially vital maxillary central incisor. After access cavity preparation, partial pulpotomy in molar and full pulpotomy in central was performed, and the remaining pulps was capped with either Biodentine or CEM cement, in each tooth. The crowns were restored with composite filling material at the following visit. The post-operative radiographic and clinical examinations (approx. average of 16 months) showed that both treated teeth remained functional, with complete root development and apex formation. A calcified bridge was produced underneath the capping material. No further endodontic intervention was necessary. Considering the healing potential of immature vital pulps, the use of CEM cement and Biodentine for apexogenesis might be an applicable choice. These new endodontic biomaterials might be appropriate for vital pulp therapies in an immature tooth. However, further clinical studies with longer follow-up periods are recommended.

## Introduction

Vital pulp therapy (VPT) of immature teeth is performed to encourage physiological development and formation of the root end and apical closure; this procedure is also referred to as apexogenesis [1, 2]. The aim of apexogenesis is the preservation of vital healthy pulp tissue so that continued root development with apical closure occurs [3]. Historically, calcium hydroxide used to be the material of choice for VPT [4]. Later, upon introduction of mineral trioxide aggregate (MTA), this bioactive material became the gold standard [5]. Recently some other calcium-silicate based cements from this aggregate were invented such as calcium-enriched mixture (CEM) cement and Biodentine that can be favorable VPT agents. 

CEM cement (Yektazist Dandan, Tehran, Iran) was introduced as an endodontic filling biomaterial. The significant components of the cement powder are calcium oxide (CaO), sulfur trioxide (SO3), phosphorous pentoxide (P2O5), and silicon dioxide (SiO2) [6].

The physical properties of CEM cement such as flow, film thickness and primary setting time are favorable and its clinical applications are similar to those of MTA [7]. CEM cement has shown similar sealing ability as MTA as retrograde filling materials in many *in vitro* studies. Animal studies have shown that PDL regeneration, cementogenesis and dentinogenesis occur in contact with CEM cement as well [8]. CEM cement is also capable to stimulate dentinal bridge formation [9].

Overall, comparing radiographic outcomes of VPT using CEM and MTA, apexogenesis occurred in 76.8% and 73.8% in CEM cement and MTA groups respectively, with no significant differences [10]. However, further clinical studies may be required.

**Figure. 1:**
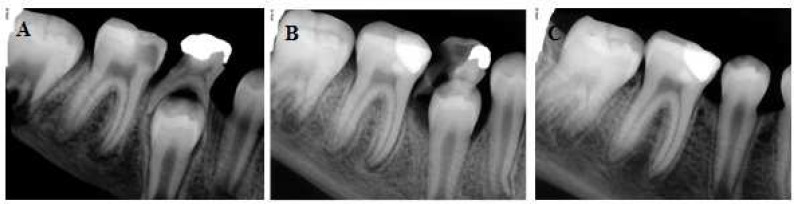
*A)* Preoperative image of the mandibular right molar with involved vital pulp and immature apices; *B*) post-operative radiographic image of the tooth after performing VPT using Biodentine; *C*) Follow-up radiography and apical maturation

Biodentine (Septodont, Saint-Maur-des-Fosses, France) is another calcium-silicate based material that has recently gained popularity and has been advocated to be used in various clinical applications. Biodentine is a bioactive inorganic calcium silicate-based cement that increases biomineralization and pulp cell proliferation [11]. The Biodentine consists of powder and liquid components. The powder contains tricalcium silicate, dicalcium silicate, and calcium oxide. The liquid consists of calcium chloride and a carboxylate-based hydro-soluble polymer that serves as the water-reducing agent. Its prominent features include easy handling and a short setting time [12]. 

In clinical settings, Biodentine has shown a similar efficacy to MTA and can be considered as an alternative for pulp capping procedures. Complete dentinal bridge formation and absence of an inflammatory response were reported [13].

The present manuscript, describes the report of two VPT cases on immature permanent teeth using both aforementioned biomaterials with long follow-up periods.

## Case Report

***Case 1*** *:* An 8 -year old girl was referred to Endodontic Department with the chief complaint of deep caries on the mandibular molar. No history of trauma was present and the patient was medically healthy. No spontaneous pain was reported by the patient. Intra-oral examination revealed extensive coronal caries, pain on percussion but not upon palpation on tooth #19. Radiographic examination revealed a cavitated tooth with immature apices with apical radiolucency around the mesial root of the tooth. She was diagnosed with irreversible pulpitis and symptomatic apical periodontitis of tooth #19. 

Under local anesthesia with 2% lidocaine and 1:80000 epinephrine (Darupakhsh, Tehran, Iran) and rubber dam isolation, access cavity was prepared with a diamond fissure bur followed by a partial pulpotomy with round diamond bur #2. Hemostasis was achieved by gentle placement of a moistened sterile cotton pellet over the amputated pulp. According to the instructions, Biodentine powder and liquid were mixed to achieve a creamy consistency. A 2-mm-thick layer of the cement was placed over the pulp using an amalgam carrier followed by Cavite temporary filling (ESPE-Premier, Norristown, PA, USA). Later the tooth was restored by a composite restoration.

Patient was recalled 3 weeks later for the first follow-up. Tooth was functional with no clinical signs or symptoms of pulpal disease. Formation of distal and mainly mesial root apex was evident. Clinical and radiographic examinations at 3, 6, and 18 months revealed the tooth was functional with no clinical signs or symptoms of pulpal disease. The final examination confirmed complete root development (Figure 1).

***Case 2:*** An 8 year-old boy was referred to the same department with a chief complaint of front tooth discoloration following trauma a month earlier. Intraoral examination revealed no swelling, no pain on palpation or percussion on tooth #9. The tooth was irresponsive to vitality and cavity tests. Radiographic examination showed immature apices with no apical lesion. The differential diagnosis was pulp necrosis or a vital pulp that was not responsive to tests after trauma and severance of nerve fibers. This final diagnosis was postponed to treatment session: Irreversible pulpitis which was not responsive to tests.

Under local anesthesia with 2% lidocaine and 1:80000 epinephrine and rubber dam isolation access cavity was prepared. Coronal pulp was removed with a high-speed sterile round diamond bur with water cooling. Although tooth was initially irresponsive to vitality tests, following the insertion of file #50 to the length of 8 mm, bleeding was induced and presence of a vital pulp was confirmed; thus VPT was suggested. 

**Figure 2:**
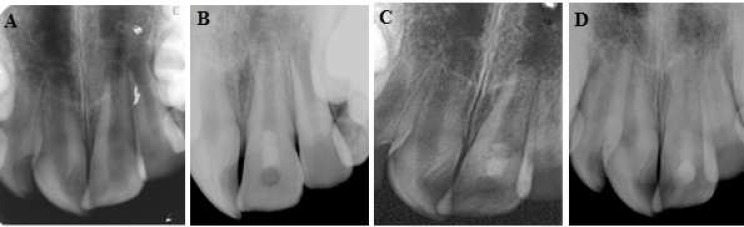
*A)* Preoperative image of the maxillary central incisor with history of trauma and immature apex, *B*) post-operative radiographic image of the tooth after performing VPT using CEM cement, *C and D*) Follow-up radiography and apical maturation

The area was rinsed with normal saline solution. Hemorrhage was controlled with placement of saline-damped sterile cotton pellet over the amputated pulp. According the instructions, CEM cement powder and liquid were mixed to achieve a creamy consistency. An approximately 2-mm thick layer of CEM was placed over the pulp using an amalgam carrier and was gently adapted to the dentinal walls of the access cavity with a dry cotton pellet. A moistened cotton pellet was placed lightly over it and followed by Cavite temporary filling. After 24 h the cotton pellet was removed and the tooth was filled with glass ionomer cement and restored with composite.

The patient was recalled within 1-3 weeks of treatment and followed for a period of 3 years on 3, 6, 9, 12 and 36 months. Apex formation and root development was evident radiographically after one year. The tooth was asymptomatic and functional. A narrow canal was observed (Figure 2).

## Discussion

VPT is the generally preferred treatment for immature teeth with vital pulps that are frequently exposed as a result of caries or trauma [14]. Numerous materials have been used for pulp capping among which calcium hydroxide and MTA are more common. MTA has been the gold standard material for use in VPT [15]. VPT is indicated to maintain pulp vitality, stimulate root development and apical closure. The goal of treatment after pulp exposure is to promote the pulp tissue healing and assist the formation of continuous layer of reparative dentin. It has been stated that the five year prospect of treatment outcome of VPT is over 95% [16].

CEM cement has been recently introduced as an applicable endodontic cement with similar clinical applications to MTA. Many desirable characteristics such as biocompatibility, integrity to pulpal tissue and positive handling characteristics have turned it to favorable cement. Considering the advantages of CEM cement over MTA, especially its improvement in tooth discoloration issue, handling and bactericidal effects, CEM cement might be an appropriate biomaterial to be used in VPT in open apex teeth [17]. It sets in aqueous environments, has excellent antibacterial activity and a short setting time (less than 1 h). In addition, CEM cement has a sealing ability similar to MTA [18].

Since healing occurs without any endodontic intervention, it is considered that CEM cement has the acceptable criteria to be used as a pulp capping material in various clinical applications.

Advantages of Biodentine over MTA are shorter setting time, better compressive strength and sealing ability. Some of its improvements compared to other calcium-silicate materials are its bioactive properties and hard tissue regeneration capability. It has been further suggested that the material had the ability to maintain a successful marginal integrity due to the formation of hydroxyapatite crystals at the surface which enhances the sealing ability. Due to this superior sealing potential, there is almost no risk of microleakage [12, 19].

It’s been stated that a particular feature of Biodentine is its capacity to continue improving in terms of compressive strength with time until reaching a similar range with natural dentine. In the study by Grech *et al*. [20], Biodentine revealed the highest compressive strength compared to the other tested materials. This result has been endorsed to its improved strength due to the low water cement ratio.

The main goal in treating immature teeth is to maintain pulp vitality so that apexogenesis can occur. The most significant indicator for success of VPT in immature permanent teeth is radiographic confirmation of root development and apex closure [7]. In this report, use of CEM cement as an artificial apical barrier showed successful clinical and radiographic outcomes in an average follow-up time of 16 months. In the presented cases, apexogenesis treatment with CEM cement and Biodentine are completed resulting in apical closure. Regular and longer follow-ups are suggested.

Favorable treatment outcomes have been achieved in VPT with calcium-silicate based materials in teeth diagnosed with irreversible pulpitis [1, 2]. Root canal treatment is considered disadvantageous due to the vast tooth structure and pulp tissue removal. Recently authors suggest that following VPT, inflamed pulps with irreversible pulpitis have a high chance to return to healthy and functional conditions [21]. This case report confirms the success of VPT with Biodentine and CEM cement in teeth with irreversible pulpitis

## Conclusion

The use of CEM cement and Biodentine seem to be valid choices in VPT cases. The authors believe that they might be appropriate biomaterials as pulp capping materials in VPT of immature permanent teeth with irreversible pulpitis. However, more clinical studies with longer follow-up periods are required.

## References

[1] Yazdani S, Jadidfard MP, Tahani B, Kazemian A, Dianat O, Alim Marvasti L (2014). Health Technology Assessment of CEM Pulpotomy in Permanent Molars with Irreversible Pulpitis. Iran Endod J.

[2] Asgary S, Fazlyab M, Sabbagh S, Eghbal MJ (2014). Outcomes of different vital pulp therapy techniques on symptomatic permanent teeth: a case series. Iran Endod J.

[3] Ingle JI (2008). Ingle's endodontics 6.

[4] Banava S, Fazlyab M, Heshmat H, Mojtahedzadeh F, Motahhary P (2015). Histological Evaluation of Single and Double-visit Direct Pulp Capping with Different Materials on Sound Human Premolars: A Randomized Controlled Clinical Trial. Iran Endod J.

[5] Maria de Lourdes RA, Holland R, Reis A, Bortoluzzi MC, Murata SS, Dezan E, Souza V, Alessandro LD (2008). Evaluation of mineral trioxide aggregate and calcium hydroxide cement as pulp-capping agents in human teeth. J Endod.

[6] Asgary S, Shahabi S, Jafarzadeh T, Amini S, Kheirieh S (2008). The properties of a new endodontic material. J Endod.

[7] Harandi A, Forghani M, Ghoddusi J (2013). Vital pulp therapy with three different pulpotomy agents in immature molars: a case report. Iran Endod J.

[8] Tabarsi B, Parirokh M, Eghbal M, Haghdoost A, Torabzadeh H, Asgary S (2010). A comparative study of dental pulp response to several pulpotomy agents. Int Endodontic J.

[9] Asgary S, Ehsani S (2009). Permanent molar pulpotomy with a new endodontic cement: A case series. J Conserv Dent.

[10] Nosrat A, Seifi A, Asgary S (2013). Pulpotomy in caries‐exposed immature permanent molars using calcium‐enriched mixture cement or mineral trioxide aggregate: a randomized clinical trial. Int J Paediat Dent.

[11] Khoshkhounejad M, Shokouhinejad N, Pirmoazen S (2015). Regenerative Endodontic Treatment: Report of Two Cases with Different Clinical Management and Outcomes. J Dent (Teh).

[12] Malkondu Ö, Kazandağ MK, Kazazoğlu E (2014). A review on biodentine, a contemporary dentine replacement and repair material. BioMed Res Int.

[13] Nowicka A, Lipski M, Parafiniuk M, Sporniak-Tutak K, Lichota D, Kosierkiewicz A, Kaczmarek W, Buczkowska-Radlińska J (2013). Response of human dental pulp capped with biodentine and mineral trioxide aggregate. J Endod.

[14] Witherspoon DE (2008). Vital pulp therapy with new materials: new directions and treatment perspectives—permanent teeth. J Endod.

[15] Torabinejad M, Chivian N (1999). Clinical applications of mineral trioxide aggregate. J Endod.

[16] Ghoddusi J, Forghani M, Parisai I (2013). New approaches in vital pulp therapy in permanent teeth. Iran Endod J.

[17] Asgary S, Eghbal MJ, Parirokh M (2008). Sealing ability of a novel endodontic cement as a root‐end filling material. J Biomed Mat Res Part A.

[18] Ghajari MF, Jeddi TA, Iri S, Asgary S (2010). Direct pulp-capping with calcium enriched mixture in primary molar teeth: a randomized clinical trial. Iran Endod J.

[19] Rajasekharan S, Martens L, Cauwels R, Verbeeck R (2014). Biodentine™ material characteristics and clinical applications: a review of the literature. Eur Arch Paediatr Dent.

[20] Grech L, Mallia B, Camilleri J (2013). Investigation of the physical properties of tricalcium silicate cement-based root-end filling materials. Dent Mater.

[21] Yazdani S, Jadidfard M-P, Tahani B, Kazemian A, Dianat O, Marvasti LA (2013). Health technology assessment of CEM pulpotomy in permanent molars with irreversible pulpitis. Iran Endod J.

